# *Plasmodium falciparum* isolate with histidine-rich protein 2 gene deletion from Nyala City, Western Sudan

**DOI:** 10.1038/s41598-020-69756-8

**Published:** 2020-07-30

**Authors:** Mohammed A. Boush, Moussa A. Djibrine, Ali Mussa, Mustafa Talib, A. Maki, Abdulrahman Mohammed, Khalid B. Beshir, Zeehaida Mohamed, Khalid Hajissa

**Affiliations:** 1 Malaria, Centre for Disease Control, Malaria, Schistosomiasis and Leishmaniasis Control Program, State Ministry of Health, Nyala, Sudan; 2 Department of Biology, Faculty of Exact and Applied Sciences, University of N’djamena, N’djamena, Chad; 3 Department of Biology, Faculty of Education, Omdurman Islamic University, Omdurman, Sudan; 4 Genetics and Molecular Biology Laboratory, Department of Zoology, Faculty of Science, University of Khartoum, Khartoum, Sudan; 5 Department of Zoology, Faculty of Science and Technology, Omdurman Islamic University, Omdurman, Sudan; 6 Department of Molecular Genetics, Institute of Molecular Biology, University of Nyala, Nyala, Sudan; 7 National Public Health Reference Laboratory, Ministry of Health, Mogadishu, Somalia; 8 Faculty of Infectious Diseases, London School of Hygiene and Tropical Medicine, London, UK; 9 Department of Medical Microbiology & Parasitology, School of Medical Sciences, Universiti Sains Malaysia, 16150 Kubang Kerian, Kelantan Malaysia

**Keywords:** Microbiology, Molecular biology

## Abstract

In remote areas of malaria-endemic countries, rapid diagnostic tests (RDTs) have dramatically improved parasitological confirmation of suspected malaria cases, especially when skilled microscopists are not available. This study was designed to determine the frequency of *Plasmodium falciparum* isolates with histidine-rich protein 2 (*pfhrp2*) gene deletion as one of the possible factors contributing to the failure of PfHRP2-based RDTs in detecting malaria. A total of 300 blood samples were collected from several health centres in Nyala City, Western Sudan. The performance of PfHRP2-based RDTs in relation to microscopy was examined and the PCR-confirmed samples were investigated for the presence of *pfhrp2* gene. A total of 113 out of 300 patients were *P. falciparum* positive by microscopy. Among them, 93.81% (106 out of 113) were positives by the PfHRP2 RDTs. Seven isolates were identified as false negative on the basis of the RDTs results. Only one isolate (0.9%; 1/113) potentially has *pfhrp2* gene deletion. The sensitivity and specificity of PfHRP2-based RDTs were 93.81% and 100%, respectively. The results provide insights into the *pfhrp2* gene deletion amongst *P. falciparum* population from Sudan. However, further studies with a large and systematic collection from different geographical settings across the country are needed.

## Introduction

Malaria remains a major public health problem particularly in sub Saharan Africa in which majority of the populations are at risk^1^. Late diagnosis and treatment of the disease can lead to life threatening conditions^2^. The World Health Organization (WHO) implements vector control, early diagnosis and prompt treatment strategies to eliminate malaria^3^. Accordingly, the early, rapid and accurate diagnosis is an essential component in malaria control and elimination programs. Microscopic detection of malaria parasites is still considered the gold standard for diagnosis of the disease; this method is relatively sensitive and allows parasitic quantitatification and species identification^4,5^. However, in remote areas, this procedure may not be easily available due to the requirement of high-quality equipment and the challenge in maintaining well-trained microscopists^6^. Alternatively, WHO recommends the use of rapid diagnostic tests (RDTs), which have later become an alternative way of establishing the rapid diagnosis of malaria infections^7^. Accordingly, RDTs were implemented as part of malaria case management along with microscopy for diagnosing malaria among suspected cases in Sudan^8^.

Most of the current malaria RDTs target the histidine-rich protein-2 (HRP2), which is a species-specific antigen of *Plasmodium falciparum*^9^. Recent reports indicate that PfHRP2-based RDTs may provide false negative results^10^. Though among the causes of false negative results are product quality, transportation, storage distribution, parasite density and due to the existence of *P. falciparum* isolates that lack the *pfhrp2* gene. Such deletions have been reported in a broader range of malaria-endemic countries in Africa^11–13^, South America^14,15^ and Asia^16^.

Recent evidence suggested that *P. falciparum* isolates with *pfhrp2* gene deletion also circulate in the neighbouring countries^17,18^ with more than 80% of microscopically confirmed isolates found to carry *pfhrp2* gene deletion in Eritrea^19^. Recently, *P. falciparum* isolates with deleted *pfhrp2* was also reported in UK travellers from Sudan and south Sudan^11^. This condition highlights the importance of molecular surveillance to detect these deletions because they could lead to false-negative diagnoses when using PfHRP2-based malaria RDTs^20^.

Despite the diagnostic threat posed by *P. falciparum* parasites lacking the *pfhrp2* gene, sporadic data is available about the existence of *P. falciparum* parasites with *pfhrp2* deletion in Sudan, which has high burden of malaria in the region. Screening of *P. falciparum* parasites with *pfhrp2* deletions in Sudan is essential to asses RDTs performance and the impact on clinical case management and malaria elimination efforts. Therefore, the present study aimed to investigate the *pfhrp2* gene deletion in *P. falciparum* isolates in Nayala City, Western Sudan.

## Results

A total of 300 suspected patients with malaria were screened for malaria parasite by microscopy; 113 patients were positive for *P. falciparum* infection. Subsequently, the microscopically confirmed specimens were tested against HRP2-based malaria RDTs. Amongst the isolates, the PfHRP2 RDTs identified 106 (93.81%) positives. A total of 7 out of 113 of *P. falciparum* microscopy-confirmed cases were RDT negative. This finding resulted in overall 93.81% [95% confidence interval (CI) 87.65–97.47] and 100% (95% CI 98.05–100) sensitivities and specificities of the PfHRP2-based RDTs respectively (Table 1). The parasite density of the seven RDT-negative specimens was 96–5,120 asexual parasites/µl with a mean of 1,540 parasites/µl.

**Table 1:** RDT performance for the diagnosis of malaria compared to microscopy as the gold standard.

	RDTs		
	Positive, n (%)	Negative, n (%)	Total
**Microsopy**			
Positive, n (%)	106 (100)	7 (3.6)	113
Negative, n (%)	0 (0)	187 (96.4)	187
Total	106	194	300

### Confirmation of *P. falciparum* infection by PCR

All the microscopy-confirmed *P. falciparum* cases were *P. falciparum* infections (113 out of 113) because all of them generated PCR products for *P. falciparum* specific ssrRNA primers. Therefore, the 106 RDT-positive isolates were found not eligible for further *pfhrp2* deletion analysis. The remaining seven isolates were further investigated to examine whether *pfhrp2* gene was lacking given the negative RDT results of these specimens.

### Detection of *Pfhrp2* gene

*Plasmodium falciparum* ssrRNA gene was amplified in all seven RDT-negative isolates. The samples were subjected to another round of PCR to amplify the flanking regions of *pfhrp2.* The *pfhrp2* gene was successfully amplified in most of the isolates (six out of seven, 85.7%), this exclude the deletion of the entire gene in all these samples. Amongst the seven samples, the entire *pfhrp2* gene was deleted in one sample (14.3%). The *pfcsp* gene was successfully amplified to confirm this deletion. In addition the parasitic density of this sample was relatively high (Table 2).

**Table 2:** Diagnostic profile and parasite density of the RDT-negative/microscopy positive isolates.

Sample ID	Results				
	RDTs	Microscopy	nPCR	HRP2	Parasiteamia (p/μl)
1	−	+	+	+	5,120
2	−	+	+	+	2,112
93	−	+	+	+	176
203	−	**+**	**+**	−	1,744
207	−	+	+	+	96
230	−	+	+	+	368
283	−	+	+	+	1,168

## Discussion

Despite the substantial drop in malaria cases and deaths over the past decade, efforts should be intensified to ensure prompt and sensitive diagnosis and effective treatment. PfHRP2-based RDTs are valuable tools for *P. falciparum* malaria diagnosis, especially in areas where routine microscopic diagnosis is inaccessible. However, studies across the globe have reported the reduced diagnostic performance of PfHRP2-based RDTs. Evidence from many African countries revealed that HRP2-based RDTs failed to detect *P. falciparum* parasites^19^. This observed failure is attributed to various factors, including the lack of the *pfhrp2* gene, which is an important factor responsible for false negative results. In Sudan, there were a few reports of false negative RDT results associated with *pfhrp2*-negative, though the availability of data regarding the *Pfhrp2* deletion in Sudanese population are limited^21^. Given that *pfhrp2*-deleted *P. falciparum* isolates have been reported in the country^11,22^, this study investigated the possible circulation of parasites with *pfhrp2* deletions in western part of Sudan.

Our study reports, for the first time, the presence of *P. falciparum* parasites with *pfhrp2* deletion in western Sudan. Of the 7 false negative RDT results, one isolate was smear-positive and *pfhrp2* PCR-negative. The presence of parasite DNA and its quality was verified by two other independent genes, suggesting that the sample has *pfhrp2* gene deletion. While the cause of false negative RDT for the 6 samples (5.3%) is unknown, 0.9% (1/113) of the false negative can be explained by *pfhrp2* deletion. The six samples determined as positive by microscopy and negative using HRP2 RDT were confirmed positive by PCR as indicated by the amplified *pfhrp2* gene. This finding could be attributed to the sequence variability among the *pfhrp2* region or low parasitaemia as some of the samples had parasitaemia below the detection limit of RDT^12,23^. Among the false negative isolates by RDT, the number of parasites density varies from 96 to 5,120 asexual parasites/µl. A very low parasite densities or target antigen concentrations could also contributed to false negative results. Another possible explanation is that other factors, including the product quality, transportation, storage and distribution, possibly affected the performance of RDTs. This is very unlikely as SD Bioline Pf/Pv/Mixed Combo cassettes used in this study was prequalified by WHO, and the shipment and storage conditions in the field were optimal and followed WHO recommendations. Thus, they are less likely to be the reasons for false negative results in this study. The most likely explanation could be low parasitaemia and/or sequence variation of the repeat region of the *pfhrp2*, which is targeted by the HRP2-based RDT.

The only one *pfhrp2* negative isolate may not reflect the actual number of the *pfhrp2* negative *P. falciparum* among the study population. This is because the 106 RDT positive isolates were not screened for *pfhrp2* gene deletion. The RDT positive signal could be generated from HRP3 due to the cross reactivity of HRP3 antigen with the HRP2. In this context, the actual number of *pfhrp2* deletion could be higher than the estimated prevalence. Nevertheless, whether the deletion of *pfhrp2* is compensated by the presence of HRP3 could not be determined and this is one of the limitations of the study.

Limited information is available regarding the deletions of *pfhrp2* gene in samples obtained from Sudanese patients. A similar recent study with a small sample size conducted by our group in the centre of Sudan reported genetic variations in *pfhrp2* and *pfhrp2*-negative samples but deletion was not confirmed^21^. Our current study, however, found one deletion that caused RDT negative result. Study area, sample size and collection period could explain the differences in findings. Other studies also reported genetic variation and suspected deletion of *pfhrp2* gene^22^ as well as *pfhrp2* deletions in malaria patients among UK travellers from Sudan^11^, though it is not clear which part of Sudan the UK patient travelled from.

To date, parasites lacking the *pfhrp2* gene have been unequivocally identified in many malaria endemic regions^16^. Despite the low prevalence of *pfhrp2* deletion in the current study, the rates of deletions vary considerably in Africa and globally. Remarkably high rates of *pfhrp2* gene deletions were reported from neighbouring Eritrea^17^. Moreover, findings from several countries indicated that *P. falciparum* isolate with *pfhrp2* gene deletion is circulating in east Africa, including Kenya^18^, Tanzania and Uganda^24^, South Sudan^11^ and Rwanda^25^. By contrast, reports of presence of *pfhrp2* gene deletions in West Africa have been limited, including in Mali^12^ and Senegal^13^. Recent studies provide evidence of deletion of the *pfhrp2* gene as reported from Equatorial Guinea^26^ and Nigeria^27^. However, in many highly malaria-endemic countries, data about *pfhrp2* deletion are missing. This underscores the importance of conducting comprehensive molecular surveillance to investigate the *pfhrp2* gene deletion in all endemic countries.

The study was not designed to systematically identify *pfhrp2* deletion in western Sudan and the prevalence does not reflect the true prevalence in the whole region. However, the prevalence of *pfhrp2* deletion is considerably lower than 5%, which is the minimum threshold needed to change the RDT types based on WHO guidelines. Evidently, malaria RDTs tests that rely on PfHRP2 for the detection of *P. falciparum* infections are still suitable for *P. falciparum* malaria diagnosis in Sudan.

Despite the importance of monitoring the deletion of *pfhrp2* gene across the whole country, this study was limited by the collection of samples from a single location in the western part of Sudan. The research needs to be expanded to cover other geographic regions given that this finding may not be representative of the situation in the whole country. Moreover, screening of *pfhrp2* gene as well as the *pfhrp3* gene among RDTs positive isolates could help in determining the actual number of HRP gene deletion among the study population, which is one of the limitations of the current study.

In conclusion, this study revealed that *P. falciparum* isolates with *pfhrp2* gene deletion are present in the parasite populations in western Sudan. The PfHRP2-based RDTs used in this study detected the majority of *P. falciparum* infections. Further studies, including a large and systematic collection from different geographical settings across the country, will be essential to estimate the *pfhrp2* deletion prevalence and to better assess the performance of *Pf*HRP2-based RDTs.

## Methods

### Study population and site

A total of 300 suspected patients with malaria from 4 health centres in Nyala City were enrolled in a cross-sectional study conducted between July 2018 and April 2019. Nyala is the capital of the South Darfur State, Western Sudan and covers an area of about 127,300 km^2^. The city lies in a savannah zone between 9° 30′ 13″ N latitude and 15° 27′ 28″ E longitude. Patients presented with symptoms consistent with uncomplicated malaria attending the selected health centres were enrolled and blood smears for Malaria were obtained from each patient.

### Sample collection

A total of 300 venous blood samples (3 ml each) were collected from each participant for the preparation of thin and thick blood films and for rapid diagnostic tests (RDTs) and for DNA extraction. An aliquot of blood was saved on a Whatman 3MM filter paper (Whatman, Clifton, NJ, USA) for parasite specimen preservation.

### Microscopy and RDT analyses

Thick and thin blood smears were prepared and stained with 10% Giemsa for 15 min. Subsequently, the stained slides were examined under a 100 × oil immersion microscope by two independent expert microscopists (conducted by a senior laboratory technician in malaria reference laboratory in the state ministry of health). Parasitaemia was calculated by counting the number of parasites observed per 200 leukocytes and assuming a total of 8,000 leukocytes/µl. Simultaneously, all blood samples were assayed with SD BIOLINE Malaria Ag P.f/P.v RDT, which is a qualitative and differential test for the immunological detection of HRP2 and parasite lactate dehydrogenase of *P. falciparum *and *P.* *vivax*, respectively. The RDT assays were performed, and the results were interpreted according to the instructions provided by the manufacturers.

### DNA extraction

For molecular analysis, genomic DNA was extracted from blood samples by using a Geneius™ Micro gDNA Extraction kit (Geneaid Biotech Ltd., Taiwan) in accordance with the manufacturer’s instructions. DNA was resuspended in 100 µl elution buffer and stored at − 20 °C.

### Confirmation of *P. falciparum* infection by polymerase chain reaction (PCR)

The presence of *P. falciparum* infection was further confirmed by species-specific nested PCR by using a small subunit ribosomal RNA (ssrRNA) gene via a previously described two-step procedure^28^. *Plasmodium*-specific primers were first used, and the PCR product of the positive samples was used as a template in a multiplex PCR system through species-specific primers.

### Detection of *pfhrp2* gene

Microscopic and PCR positive *P. falciparum* samples but RDT negative were subjected to *pfhrp2* PCR amplification. Specific primers *Pfhrp*2-F (5′ ATTCCGCATTTAATAATAACTTG TGTAGC 3′) and *Pfhrp*2-R (5′ ATGGCGTAGGCAATGTGTGG 3′) targets exon 2 of *pfhrp2* were used as described previously^12^.

The PCR mixture had a total volume of 20 µl containing 4 µl of 5 × HOT FIREPol Blend Master Mix, 0.75 µM each primer and 2 µl DNA. The thermoprofile process consisted of an initial denaturation step at 95 °C for 4 min, followed by 35 cycles at 95 °C for 30 s, 58 °C for 45 s, 72 °C for 1 min and a final extension step at 72 °C for 5 min. Furthermore, the samples in which no *pfhrp2* was amplified were subjected to *P. falciparum* circumsporozoite gene (*pfcsp*) PCR amplification, with the assumption that successful ssrRNA and *csp* gene amplification indicates reasonable quantity and quality of DNA that would allow *pfhrp2* amplification. Thus, all the samples that were not amplified by *pfhrp2* PCR may have *pfhrp2* gene deletion.

### Ethical statement

This study protocol was reviewed and approved by the Institute of Molecular Biology, University of Nyala and the committee of the Research directorate, Federal Ministry of Health (fmoh/nhrc/rd/rec). Prior to blood samples collection, written informed consent was obtained from all subjects and also from a parent or guardian for children less than 18 years of age. All methods were performed in accordance with the relevant guidelines and regulations.

## Data Availability

Any further requested information regarding the experimental and data analysis during the current study is available from the corresponding author on reasonable request.
